# Enhanced expression of the central survival of motor neuron (SMN) protein during the pathogenesis of osteoarthritis

**DOI:** 10.1111/jcmm.12170

**Published:** 2013-11-17

**Authors:** Magali Cucchiarini, Henning Madry, Ernest F Terwilliger

**Affiliations:** a Center of Experimental Orthopaedics, Saarland University Medical CenterHomburg/Saar, Germany; b Department of Orthopaedics and Orthopaedic Surgery, Saarland University Medical CenterHomburg/Saar, Germany; c Harvard Institutes of Medicine Beth Israel Deaconess Medical Center, Harvard Medical SchoolBoston, MA, USA

**Keywords:** osteoarthritis progression, pro-inflammatory cytokines, SMN expression

## Abstract

The identification of new components implicated in the pathogenesis of osteoarthritis (OA) might improve our understanding of the disease process. Here, we investigated the levels of the survival of motor neuron (SMN) expression in OA cartilage considering the fundamental role of the SMN protein in cell survival and its involvement in other stress-associated pathologies. We report that SMN expression is up-regulated in human OA compared with normal cartilage, showing a strong correlation with the disease severity, a result confirmed *in vivo* in an experimental model of the disease. We further show that the prominent inflammatory cytokines (IL-1β, TNF-α) are critical inducers of SMN expression. This is in marked contrast with the reported impaired levels of SMN in spinal muscular atrophy, a single inherited neuromuscular disorder characterized by mutations in the *smn* gene whereas OA is a complex disease with multiple aetiologies. While the precise functions of SMN during OA remain to be elucidated, the conclusions of this study shed light on a novel pathophysiological pathway involved in the progression of OA, potentially offering new targets for therapy.

## Introduction

Osteoarthritis (OA) is a degenerative disease of the whole joint 1 that affects millions of people worldwide. Osteoarthritis is mainly characterized by a slow, progressive destruction of major components of the extracellular cartilage matrix, resulting from a disturbed balance of physiological processes in chondrocytes 2, the unique cartilage-forming cells. Various risk factors have been associated with the incidence of OA (genetic background, ageing, trauma, obesity and metabolic conditions) 3–6. Several lines of evidence have demonstrated the critical involvement of stress stimuli in the pathogenesis of OA, including mechanical stress (loading), oxidative and biochemical stress (production of nitric oxide and of reactive oxygen species; release of cartilage matrix degradation products, of pro-inflammatory cytokines like the prominent interleukin 1 beta - IL-1β and tumour necrosis factor alpha - TNF-α, and of matrix-degrading enzymes) 7–8. It is well accepted that chondrocytes are key players in the initiation and progression of OA. In normal adult cartilage, they are terminally differentiated cells showing basically no proliferative activity and maintaining the matrix components (proteoglycans, type-II collagen) under low turnover. In early OA, the chondrocyte undergo significant changes in their gene expression profiles (including through epigenetic modifications) 9–10, leading to transient proliferative responses and to the synthesis of molecules that are not naturally present in the adult cartilage (type-X, type-III, type-VI, and type-IIA collagen, tenascin, decorin) while stopping to express the major matrix components 10–11. This has been seen as an attempt at repair but that is eventually counterbalanced by the activation of stress pathways and molecules, by a decline in the cell responsiveness to reparative stimuli, and by cell senescence and degeneration, leading to an irreversible deterioration of the cartilage architecture 12.

Despite recent, critical advances in the identification of OA-related biomarkers that might allow for rapid and effective early OA diagnosis (and for a potential development of beneficial treatment options) 13–14, the pathophysiology of this complex disorder is not yet fully understood 15,16, showing the need to explore new aspects or components of the disease that might define new targets for therapy. As chondrocytes are subjected to various stress stimuli in the OA joint, we investigated the expression profiles of the *survival of motor neuron* (*smn*) gene in the cartilage during the course of OA in light of the response of the SMN product to stress conditions (environmental stress, heat shock and irradiation) by accumulation of the molecule in stress granules 18 and its significant role in oxidative stress 18,19. Survival of motor neuron is a central, ubiquitously expressed protein part of macromolecular complexes, that is of fundamental importance for the survival of all eukaryotic cells 21 and displaying housekeeping functions in RNA metabolism (biogenesis of RNA-protein complexes, pre-mRNA splicing) 22. For the first time, we report that the levels of SMN are up-regulated in human OA compared with normal adult cartilage, showing a strong correlation with the disease severity and an influence of key OA mediators (IL-1β and TNF-α), an observation confirmed by analysing the SMN levels in a model of experimentally induced OA *in vivo*. As SMN has further been reported for its anti-apoptotic activities 23 and its influence on transcriptional and post-transcriptional gene regulation 24–25, all processes relevant of the pathogenesis of OA 12,26, active work is ongoing to gain insights into the specific roles of differential SMN cartilage expression in OA cartilage and to address the question whether SMN up-regulation is a protective response or is causally involved in this disease.

## Materials and methods

### Reagents and antibodies

All reagents were from Sigma-Aldrich (Munich, Germany) unless otherwise indicated. The recombinant human IL-1β and human TNF-α were purchased at R&D Systems (Wiesbaden-Nordenstadt, Germany). The anti-SMN antibodies (H-195 and 2B1) were from Santa Cruz Biotechnologies (Santa Cruz, CA, USA).

### Subjects and chondrocyte culture

Normal human adult articular cartilage was obtained from unaffected knee joints of patients (*n* = 13; ages 67–72) that underwent tumour surgery. Osteoarthritic cartilage was obtained from the joints of patients (*n* = 52; ages 65–78) undergoing total knee arthroplasty. All patients were from the Department of Orthopaedics and Orthopaedic Surgery of the Saarland University Medical Center, Homburg, Germany. The study was approved by the Ethics Committee of the Saarland Physicians Council. All patients provided informed consent before inclusion in the study. Research was in compliance with the World Medical Association Declaration of Helsinki. Cartilage explants (6.2-mm diameter) and articular chondrocytes were prepared and isolated immediately after collection as previously described 28. Explants and cells were cultured in DMEM, 50 μg/ml ascorbic acid, 100 U/ml penicillin G, 100 μl/ml streptomycin containing 10% foetal bovine serum (FBS) in a humidified atmosphere with 10% CO_2_ at 37°C. Cells were tested after only one passage in culture to avoid the shift in chondrocyte phenotype. Some parts of all the normal cartilage explants as well as cells freshly isolated from this explants were also incubated with IL-1β or TNF-α (each at a concentration of 10 or 100 ng/ml) for 10 days at 37°C with 10% CO_2_, with medium change every 3 days for further analyses.

### Animals and experimental osteoarthritis

Thirteen mature chinchilla bastard rabbits (3.5 ± 0.5 kg weight, 7–8 months old, with closed epiphyses and absence of joint pathology; Charles River, Sulzfeld, Germany) were employed for the study. All procedures were approved by the Saarland University Animal Committee according to German guidelines. Seven rabbits underwent surgical anterior cruciate ligament transection (ACLT) to induce experimental OA lesions in the knee joint cartilage. Using a medial parapatellar approach, the patella was dislocated laterally and the knee placed in full flexion. The ACL was exposed and transected with a #15 scalpel blade. Complete transection was confirmed by a positive intra-operative Lachman test. The incisions were then closed in layers. Post-operatively, the rabbits were allowed normal cage activity without immobilization. Six other rabbits that were sham operated (arthrotomy only) served as controls. The femorotibial joints of the thirteen animals were removed 4 weeks after surgery 29 for further analyses. Isolation of articular chondrocytes was also performed as described above.

### Histological analysis

Cartilage explants were processed and paraffin-embedded sections (5 μm) were stained with safranin O to detect proteoglycans and with haematoxylin and eosin to detect cells 28. The severity of OA changes was histomorphologically graded on safranin O/haematoxylin and eosin-stained sections using the scoring method described by Mankin *et al*. 30. This grading system is based on a semi-quantitative evaluation of the cartilage as follows: structure (0: normal; 1: surface irregularities; 2: pannus and surface irregularities; 3: clefts to the transitional zone; 4: clefts to the radial zone; 5: clefts to the calcified zone; 6: complete disorganization), cells (0: normal; 1: diffuse hypercellularity; 2: cloning; 3: hypocellularity), safranin O staining (0: normal; 1: slight reduction; 2: moderate reduction; 3: severe reduction; 4: no dye noted) and tidemark integrity (0: intact; 1: crossed by blood vessels).

### Immunohistochemical, immunocytochemical and histomorphometric analyses

The SMN protein was detected on histological sections of cartilage using specific antibodies, a biotinylated secondary antibody (Vector Laboratories, Grünberg, Germany), and the avidin-biotin-peroxidase method (Vector Laboratories) with diaminobenzidine as the chromogen 28. To control for secondary IgGs, sections were processed with omission of the primary antibody. Samples were examined under light microscopy (BX45 microscope; Olympus, Hamburg, Germany). The percentage of cells positive for SMN immunoreactivity were measured at three standardized sites or using 10 serial immunohistochemical sections for each parameter, test and replicate condition. Analysis programs included SIS AnalySIS (Olympus), Adobe Photoshop (Adobe Systems, Unterschleissheim, Germany) and Scion Image (Scion Corporation, Frederick, MD, USA). For immunocytochemical analyses, freshly isolated chondrocytes (2 × 10^4^) were fixed, incubated with anti-SMN antibodies and a Texas-Red-conjugated secondary antibody (Vector Laboratories) 28. To control for secondary IgGs, cells were processed with omission of the primary antibody. Fluorescence was examined under a fluorescent microscope (CKX41; Olympus) using a 580-nm filter.

### Biochemical analyses of the SMN protein

All cartilage explants were processed and analysed for Western blotting analyses as previously described using 50 μg proteins 28. Expression was revealed with specific antibodies, horseradish peroxidase-labelled secondary antibodies (Vector Laboratories), and the ECL Advance Western blotting detection kit (Amersham Biosciences, Freiburg, Germany). The SMN contents in freshly isolated chondrocytes were measured by an SMN immunoassay as described by Kolb *et al*. 31 with minor modifications. Briefly, chondrocytes (10^4^) were adhered to the bottom of the wells of 96-well plates, fixed, permeabilized with 0.1% Triton and blocked with 20% FBS. The anti-SMN 2B1 antibody (1:500) was incubated and bound antibodies were detected using a peroxidase-conjugated antibody (Vector Laboratories) and the Supersignal ELISA Femto Maximum Sensitivity Substrate (Pierce, Bonn, Germany). The luminescent intensity was measured using a GENios spectrophotometer/fluorometer (Tecan, Crailsheim, Germany). Signal background was determined for each sample by omitting the primary antibody. A recombinant SMN protein (Abnova, Taipei City, Taiwan) was employed to generate a standard curve.

### Isolation of RNA, cDNA synthesis and real-time RT-PCR

RNA was prepared from all freshly isolated chondrocytes (10^6^) using the RNeasy Protect Mini Kit (Qiagen GmbH, Hilden, Germany) with an ‘on-column’ DNase digestion. Quantification of the RNA concentrations was performed by spectrophotometry; 1 μg RNA was converted to cDNA using the 1st Strand cDNA Synthesis Kit for RT-PCR/AMV (Roche Applied Science, Mannheim, Germany) exactly according to the manufacturer’s instructions. Real-time RT-PCR was performed with 2 μl cDNA with the Brilliant SYBR Green QPCR Master Mix (Stratagene - Agilent, Waldbronn, Germany) to detect the transcripts for SMN (forward primer 5′-AAAAGAAGGAAGGTGCTCACATTC-3′ and reverse primer 5′-TGGTGTCATTTAGTGCTGCTCTATG-3′), type-II collagen (forward primer 5′-GGACTTTTCTCCCCTCTCT-3′ and reverse primer 5′-GACCCGAAGGTCTTACAGGA-3′), and GAPDH for normalization (forward primer 5′-GAAGGTGAAGGTCGGAGTC-3′ and reverse primer 5′-GAAGATGGTGATGGGATTTC-3′) (all primers at 150 nM; Invitrogen GmbH, Karlsruhe, Germany) 32–33. The reaction was carried out on a Mx3000P® QPCR System (Stratagene) for 10 min. 95°C, 40 × [30 sec. 95°C, 1 min. 55°C, 30 sec. 72°C], 1 min. 95°C, and 30 sec. 55°C. The end products were examined by gel electrophoresis. Results were captured using the MxPro™ QPCR Software (Stratagene).

### Statistical analysis

Data are expressed as mean ± SD. All human and rabbit cartilage samples were evaluated for the experiments *in situ* and *in vitro*. Data were obtained by two individuals who were blinded with regard to the groups. The *t*-test and Mann–Whitney Rank Sum Test were employed where appropriate. Statistical analysis was performed with the SPSS software (SPSS Inc./IBM, Chicago, IL, USA). *P* values < 0.05 were considered statistically significant.

## Results

### SMN expression levels increase in human OA cartilage, correlating with the disease severity

**Figure 1:**
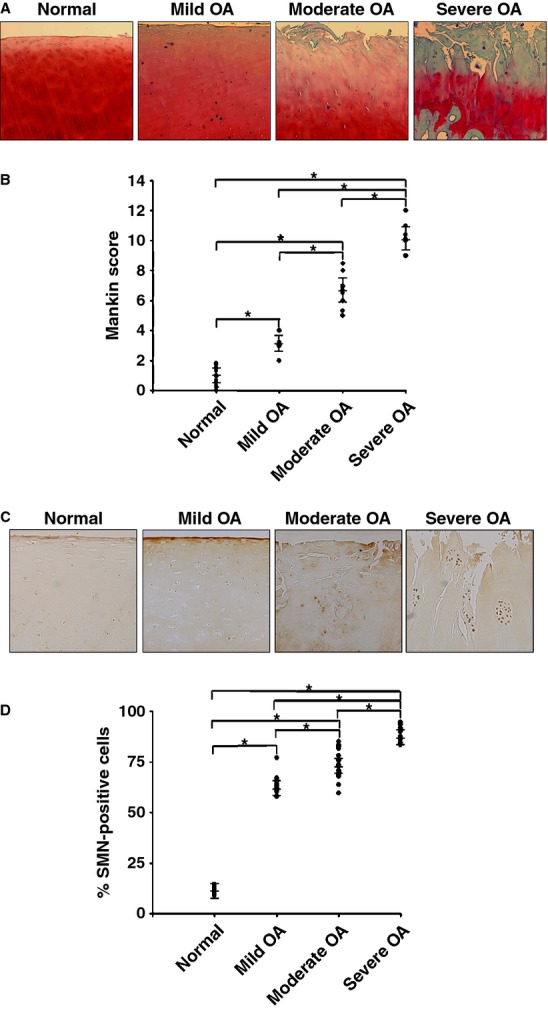
Histological grading and survival of motor neuron (SMN) expression in human normal and osteoarthritis (OA) cartilage. All samples were processed for the analysis. (A) Safranin O staining of sections from cartilage explants (representative samples; magnification ×4). (B) Grading of OA severity on safranin O-stained histological sections from all individual explants (normal donors, n = 13, Mankin score 0–2; mild OA patients, n = 14, Mankin score 2–4; moderate OA patients, n = 22, Mankin score 5–8; severe OA patients, n = 16, Mankin score 9–14). (C) SMN-specific immunoreactivity on histological sections from cartilage explants (representative samples; magnification ×10). (D) Percentages of SMN-positive cells on immunohistochemical sections from all individual explants (8.8–14.5%, 57.6–77.0%, 59.6–85.1% and 83.3–94.5% in normal, mild, moderate and severe OA samples, respectively). *Statistically significant difference between groups.

The cohort of OA patients included in the study was divided into three groups based on a histomorphometric score established by Mankin *et al*. 30 for the semi-quantitative estimation of OA extent on safranin O-stained histological cartilage sections (Fig. 1A). Accordingly, significant differences could be observed between these three groups and compared with normal donors (always *P* ≤ 0.001; Fig. 1B).

**Figure 2:**
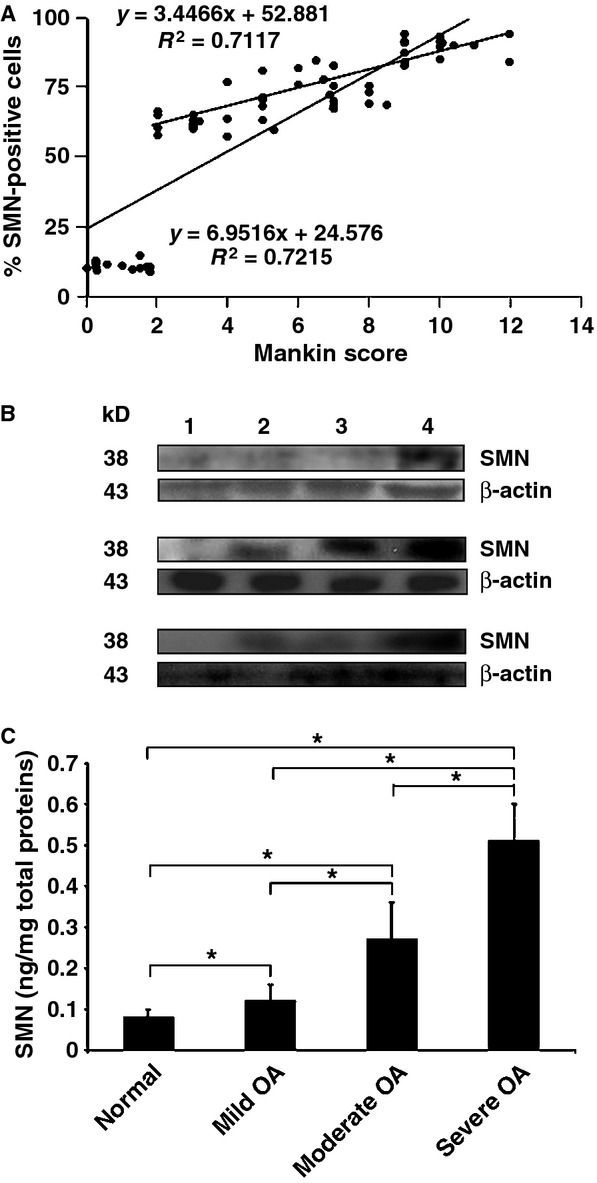
Correlation and biochemical analyses. (A) All individual values of (Fig. 1B and D) were combined to evaluate the correlation between the two parameters. (B) Western blotting analysis in human normal and osteoarthritis (OA) cartilage (1: normal cartilage, 2: mild OA, 3: moderate OA, 4: severe OA; representative samples). (C) Survival of motor neuron concentrations in freshly isolated human normal and OA chondrocytes from all the samples (0.08 ± 0.02, 0.11 ± 0.02, 0.26 ± 0.07 and 0.52 ± 0.06 ng/mg total proteins in normal, mild, moderate and severe OA samples, respectively). *Statistically significant difference between groups.

An immunohistochemical analysis of SMN was performed (Fig. 1C) to estimate the percentages of cells reactive for intracellular SMN in all the samples (Fig. 1D). Specific immunoreactivitiy was seen both in the superficial and middle zones of the cartilage. Significant increases in the levels of SMN protein were noted both in mild (up to 9.1-fold), moderate (up to 10.1-fold) and severe OA (up to 11.1-fold) relative to normal cartilage (always *P* ≤ 0.001). Also, the levels of SMN protein significantly increased in moderate (up to 1.5-fold) and severe OA (up to 1.6-fold) *versus* mild OA cartilage (always *P* ≤ 0.001) and in severe compared with moderate OA cartilage (up to 1.6-fold, *P* ≤ 0.001). When the percentages of SMN-positive cells (Fig. 1D) were combined with the corresponding, individual histological scores per donor or patient (Fig. 1B), a strong correlation was observed between the levels of SMN protein and the disease severity (*R*^2^ = 0.7215 when including normal and OA cartilage, *R*^2^ = 0.7117 with only OA cartilage, always *P* ≤ 0.001; Fig. 2A).

A Western blotting analysis of protein extracts using all the normal and OA cartilage samples demonstrated a single band of about 38 kD corresponding to the SMN protein 22–34 (Fig. 2B for representative results). In good agreement with the findings of the immunohistochemical analysis, the intensity of the SMN band was always more intense in OA relative to normal cartilage (about 1.5-, 1.6- and 5-fold difference in mild, moderate and severe OA cartilage, respectively) and increased with the disease severity (about 1.1- and 3.3-fold in moderate and severe OA *versus* mild OA, respectively and about 3.1-fold in severe compared with moderate OA).

Quantitative estimation of the amounts of SMN protein by ELISA using freshly isolated normal and OA chondrocytes from all the samples revealed a significant increase in the SMN concentrations both in mild (1.5-fold, *P* = 0.045), moderate (3.4-fold, *P* ≤ 0.001) and severe OA (6.4-fold, *P* = 0.002) relative to normal cartilage (Fig. 2C). Again, the amounts of SMN significantly increased in moderate (2.3-fold) and severe OA (4.3-fold) *versus* mild OA cartilage (always *P* ≤ 0.001) and in severe compared with moderate OA cartilage (1.9-fold, *P* ≤ 0.001).

### Increased abundance of SMN-specific bodies in human OA chondrocytes

**Figure 3:**
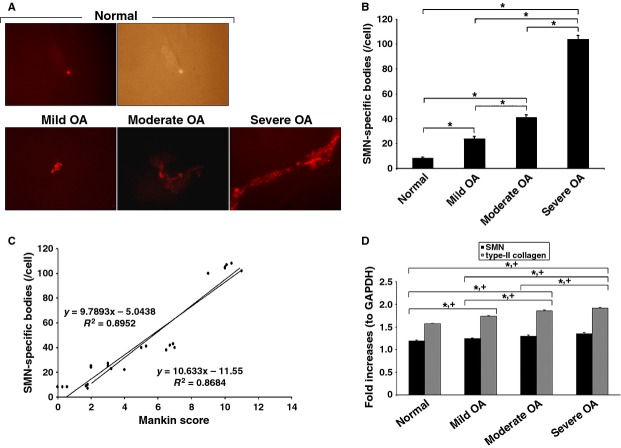
Molecular analyses. All the samples were processed for the analyses. (A) Survival of motor neuron (SMN)-specific immunoreactivity (representative samples; normal chondrocytes are also shown in regular light; magnification ×200). (B) Abundance of SMN-specific bodies. (C) Correlation between the abundance of SMN-specific bodies and osteoarthritis (OA) severity. The values of Figures 1B and 3B were combined to evaluate the correlation between the two parameters. *Statistically significant difference between groups. (D) Normalized mRNA expression of SMN (1.17–1.22, 1.24–1.27, 1.27–1.33 and 1.32–1.38 in normal, mild, moderate and severe OA samples, respectively) and type-II collagen (1.57–1.59, 1.72–1.76, 1.84–1.88 and 1.91–1.94 in normal, mild, moderate and severe OA samples, respectively). Statistically significant difference between groups for *SMN and ^+^type-II collagen.

An immunocytochemical analysis in freshly isolated normal and OA chondrocytes from all the samples further revealed that the SMN protein was present in discrete structures (Fig. 3A), consistent with previous reports identifying SMN as part of macromolecular structures (gems) in distinct cell types (muscle, spinal cord and CNS tissue, muscle, skin fibroblasts) 34–35. An estimation of the abundance of such SMN-specific bodies showed that the amounts of structures per cell significantly increased both in mild (threefold, *P* ≤ 0.001), moderate (fivefold, *P* ≤ 0.001) and severe OA (13-fold, *P* = 0.002) relative to normal cartilage (Fig. 3B). Again, the abundance of these structures significantly increased in moderate (1.7-fold) and severe OA (4.3-fold) *versus* mild OA cartilage (always *P* ≤ 0.001) and in severe compared with moderate OA cartilage (2.5-fold, *P* ≤ 0.001). When the abundance of SMN-specific bodies (shown on Fig. 3B) were combined with the corresponding, individual histological scores per donor or patient (shown on Fig. 1B), a strong correlation was observed between the amounts of structures and the disease severity of (*R*^2^ = 0.8952 when including normal and OA cartilage, *R*^2^ = 0.8684 with only OA cartilage, always *P* ≤ 0.001; Fig. 3C).

### Increased SMN mRNA levels in human OA chondrocytes

Furthermore, SMN mRNA expression was next evaluated by real-time RT-PCR analysis in cellular extracts from all freshly isolated normal and OA chondrocytes of the samples. In agreement with the findings on the protein, the levels of SMN mRNA expression increased with the disease severity and relative to normal cartilage (Fig. 3D). Accordingly, a significant increase in the intracellular ratios was noted both in mild (up to 1.09-fold, *P* = 0.005), moderate (up to 1.14-fold, *P* = 0.001) and severe OA (up to 1.18-fold, *P* ≤ 0.001) *versus* normal cartilage. Also, the ratios significantly increased in moderate (up to 1.07-fold, *P* = 0.016) and severe OA (up to 1.11-fold, *P* ≤ 0.001) compared with mild OA cartilage and in severe relative to moderate OA cartilage (up to 1.09-fold, *P* = 0.033). For comparison, the intracellular type-II collagen/GAPDH ratios were also analysed, showing a significant up-regulation with the extent of OA and *versus* normal cartilage (Fig. 3D), possibly because of an equilibration of the phenotype in the cell in contrast with the situation in the extracellular matrix compartment. A significant increase in the ratios was noted both in mild (up to 1.12-fold), moderate (up to 1.20-fold) and severe OA (up to 1.24-fold) compared with normal cartilage (always *P* ≤ 0.001). Also, the ratios significantly increased in moderate (up to 1.09-fold) and severe OA (up to 1.13-fold) relative to mild OA cartilage (always *P* ≤ 0.001) and in severe *versus* moderate OA cartilage (up to 1.05-fold, *P* = 0.002). Yet, the levels of SMN mRNA expression and fold increases noted during OA progression did not reach those attained for type-II collagen (always *P* ≤ 0.001).

### Effects of IL-1β and TNF-α upon cartilage SMN expression

**Figure 4:**
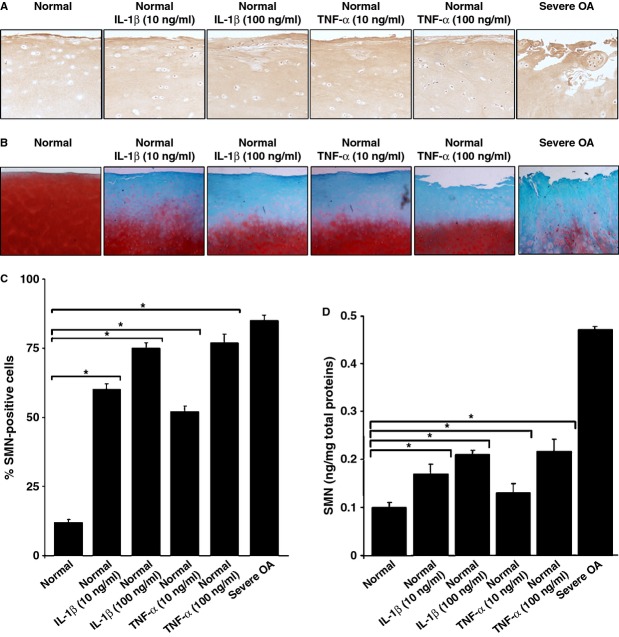
Effects of IL-1β and TNF-α upon the survival of motor neuron (SMN) expression levels in human cartilage and chondrocytes. All human normal cartilage explants and cells freshly isolated from these explants were treated for 10 days with either IL-1β or TNF-α (each at either 10 or 100 ng/ml) and processed as described in the Materials and Methods. Explants and cells defined with severe osteoarthritis (OA) were used as positive controls. (A) SMN-specific immunoreactivity on histological explant sections (representative samples; magnification ×20). (B). Safranin O staining of histological explant sections (representative samples; magnification ×2). (C) Percentages of SMN-positive cells on immunohistochemical explant sections (all samples were processed for the analysis) (normal samples: 9.1–12.7% raising at 60.2–63.1%, 73.4–76.8%, 50.8–53.4% and 77.4–78.6% with IL-1β or TNF-α at 10 or 100 ng/ml, respectively; severe OA samples: 84.1–86.4%). (D) SMN concentrations in freshly isolated chondrocytes (all samples were processed for the analysis) (normal samples: 0.10 ± 0.01 ng/mg total proteins raising at 0.18 ± 0.01, 0.22 ± 0.02, 0.14 ± 0.01 and 0.23 ± 0.01 ng/mg total proteins with IL-1β or TNF-α at 10 or 100 ng/ml; severe OA samples: 0.47 ± 0.01 ng/mg total proteins). *Statistically significant difference between groups.

As IL-1β and TNF-α have been described as key mediators of OA, we examined whether application of these cytokines induces detectable changes in the SMN immunoreactivity (%) expression profiles of human normal cartilage and freshly isolated chondrocytes (Fig. 4A and C). Treatment with IL-1β or TNF-α significantly increased the levels of SMN protein compared with normal cartilage (between 4.9- and 7.3-fold, always *P* ≤ 0.001) in a dose-dependent manner (1.2- or 1.5-fold increase with IL-1β or TNF-α from 10 to 100 ng/ml, always *P* ≤ 0.001), reaching the levels noted in mild OA when using concentrations of 10 ng/ml and those of moderate OA at 100 ng/ml. An estimation of the amounts of protein in freshly isolated chondrocytes by ELISA further showed significant, dose-dependent increases in the SMN concentrations when providing IL-1β or TNF-α at 10–100 ng/ml relative to normal, untreated cells (between 1.4- and 2.3-fold difference to the normal condition and an up to 1.6-fold increase depending on the dose, always *P* ≤ 0.001; Fig. 4D). The histological analysis of safranin O-stained sections confirmed the effects of both cytokines on cartilage matrix degradation (Fig. 4B).

### Differential SMN expression in experimental OA

**Figure 5:**
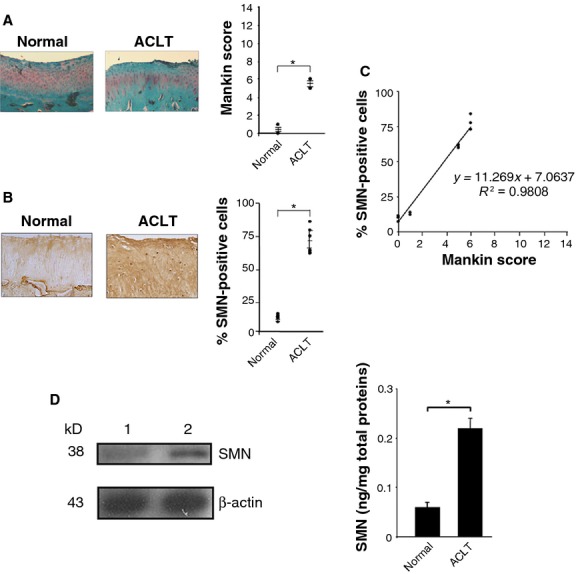
Survival of motor neuron (SMN) expression levels in an experimental osteoarthritis (OA) model *in vivo*. (A) Safranin O staining of histological sections from rabbit knee joint cartilage (representative samples; view of the femoral condyle; magnification ×4) and grading of OA severity (all samples were processed for the analyses). (B) SMN-specific immunoreactivity on sections from rabbit knee joint cartilage (representative samples; view of the femoral condyle; magnification ×20) and percentages of SMN-positive cells (all samples were processed for the analyses) (normal cartilage: 7.1–13.7%; OA cartilage: 59.7–83.8% as for moderate human OA). (C) Correlation between the SMN expression levels and OA severity (combination of all values from A and B). (D) Analysis of SMN by Western blotting (lane 1, normal cartilage; lane 2, ACLT cartilage; all representative samples) and ELISA (SMN concentrations in all freshly isolated normal and ACLT chondrocytes; *statistically significant difference).

Survival of motor neuron expression was next investigated in an *in vivo* model of OA induced by ACLT in rabbits to substantiate the results of the analysis in human articular cartilage. Four weeks after ACLT, all knees demonstrated degenerative changes on histological sections of the articular cartilage of the femoral condyles, patellar grooves and tibial plateaus *versus* normal articular cartilage from sham-operated animals (Fig. 5A) 30, in good agreement with previous findings showing that OA changes occur rapidly in this model (within 3–8 weeks) 29. The extent of OA in rabbit articular cartilage (Mankin score 5–6) was comparable to a moderate OA in human articular cartilage. Accordingly, the scores attributed to rabbit OA cartilage were significantly higher than those of normal rabbit cartilage (Mankin score 0–1; *P* = 0.001; Fig. 5A).

As noted for human cartilage, the percentages of cells immunoreactive for SMN in rabbit OA cartilage were significantly higher than those in normal rabbit cartilage (up to 11.8-fold; *P* ≤ 0.001; Fig. 5B). When these percentages were combined with the corresponding histomorphometric score per animal, a strong correlation was observed between the levels of SMN immunoreactivity and the extent of experimental OA (*R*^2^ = 0.9808, *P* ≤ 0.001) (Fig. 5C). Western blotting analysis of protein extracts from rabbit cartilage demonstrated a specific band of about 38 kD similar to that detected in human cartilage 22–34 (Fig. 5D) and that was more intense in rabbit OA cartilage compared with normal rabbit cartilage (about twofold as for moderate human OA). Quantitative estimation of the SMN protein in freshly isolated chondrocytes by ELISA showed a significant increase in the SMN concentrations in rabbit OA cartilage relative to normal rabbit cartilage (3.7-fold, *P* ≤ 0.001; Fig. 5D), as noted in moderate human OA.

## Discussion

Osteoarthritis is one of the most common disabling human conditions that affects the whole joint, leading to an irreversible destruction of the articular cartilage structure and extracellular matrix. Evidence has accumulated that the chondrocytes, the unique cartilage-forming cells, play central roles in the disease process, undergoing multiple changes in their gene expression profiles 9–10 in response to a variety of stress stimuli (loading, inflammation, oxidative stress) 7–8. Despite the identification of various biomarkers of the disease, there is a need to better characterize the key cellular and molecular mechanisms that govern the initiation and progression of OA, as the pathophysiology of this complex disorder remains poorly understood. In this study, we specifically examined the expression patterns of the product of the *smn* gene in OA cartilage in light of the implication of SMN in stress-activated pathways 18,19 that are also observed during the OA disease.

For the first time to our best knowledge, we demonstrate that the essential 38-kD cellular SMN protein is expressed both in human normal and OA cartilage in the form of discrete bodies, as previously reported in other tissues 34–35. Most remarkably, we provide evidence showing significant increases in the SMN levels in human OA *versus* normal adult cartilage, strongly correlating with the extent of the disease, a result corroborated by findings *in vivo* in a relevant model of experimental OA, suggesting a central, common role of SMN between species and confirming its importance during OA. Interestingly, while modest increases were noted at the mRNA level, more robust effects were observed at the protein level, possibly related to a build up of the protein by post-translational modifications 36. Work is ongoing to analyse the profiles of SMN in other tissues relevant of the pathogenesis of OA (synovium, subchondral bone, bone marrow compartment). Finally, we also demonstrate a dose-dependent up-regulation of the SMN levels in the presence of IL-1β and TNF-α, two prominent stress cytokines and critical mediators of the disease pathogenesis. As a matter of fact, a number of *cis*-elements responsive to disease-associated cytokines (IL-6, interferons beta and gamma - IFN-β and -γ) have been already identified in the promoter region of the *smn* gene 37, supporting the concept of SMN induction under OA-specific biochemical stress. Yet, other mediators that might also influence SMN expression should not be excluded (mechanical and oxidative stress, adipokines, epigenetics) 9,38, being currently under active investigation. Nevertheless, this study provides strong evidence of the significance of SMN regulation in OA in human damaged cartilage, a finding corroborated by the results of an analysis *in vivo*. To our best knowledge, this is the first study showing the implication of SMN during the progression of OA, shedding light on a novel cellular component of the disease that might allow to open new avenues of research on the onset of OA and to identify alternative, potent targets for therapy.

Regarding the possible roles played by SMN in OA, it is interesting to note that beside its housekeeping functions, SMN is a molecule essential for cell survival 21, displaying anti-apoptotic properties 23 possibly *via* direct interactions with Bcl-2 and p53 40–41, and having a key influence on transcriptional and post-transcriptional gene regulation 24–25. As a matter of fact, several lines of evidence have demonstrated the significance of cell death 12 and transcriptional regulation 26–27 during the course of OA. The details of the implication of SMN in these mechanisms during OA remain to be elucidated. A comprehensive analysis in experimental systems of SMN inhibition in OA cartilage 20 or of overexpression in normal cartilage 19 may provide insights into the putative functions of SMN in this disorder and address the question whether SMN up-regulation is a protective response or is causally involved in the pathogenesis of OA.

Interestingly, SMN has been originally identified as the key determinant of spinal muscular atrophy (SMA) 42, a single inherited neuromuscular disorder characterized by the degeneration of spinal motor neurons leading to muscular paralysis with muscular atrophy 43. Spinal muscular atrophy is linked to recessive mutations in the *smn* gene 44–45, showing decreased SMN concentrations in the spinal cord, skeletal muscle, liver and fibroblasts of SMA patients and a strong correlation between the disease severity and the SMN levels 34–46. It is noteworthy that we observed an up-regulation of cartilage SMN during OA whereas a dramatic reduction was demonstrated in SMA. While SMA has been identified as a single disorder linked to recessive mutations in the *smn* gene, OA represents a complex disease with multiple aetiologies (genetic background, ageing, trauma, obesity, stress environment). It is likely that the causative mechanisms regulating *smn* expression in these two pathologies are different. The results of future analyses as those described above (SMN blockade *versus* overexpression) might also help to better understand the divergence in the SMN profiles between OA and SMA. Taken together, the present findings describe SMN as a novel cellular component of the pathogenesis of OA that may provide new targets for therapy of this widespread, incurable disorder.
